# Suppression subtractive hybridization method for the identification of a new strain of murine hepatitis virus from xenografted SCID mice

**DOI:** 10.1007/s00705-015-2592-y

**Published:** 2015-09-08

**Authors:** Mohammed M. Islam, Brendan Toohey, Damian F. J. Purcell, George Kannourakis

**Affiliations:** 1 Fiona Elsey Cancer Research Institute, 106-110 Lydiard Street South, Ballarat Technology Park Central, Ballarat, VIC 3353 Australia; 2 Department of Microbiology and Immunology, Peter Doherty Institute for Infection and Immunity, The University of Melbourne, Parkville, VIC 3010 Australia; 3 School of Applied and Biomedical Sciences, Federation University Australia, Ballarat, VIC 3350 Australia

**Keywords:** SCID Mouse, Suppression Subtractive Hybridization, Subtract cDNA Library, Tester cDNA, Driver cDNA

## Abstract

During attempts to clone retroviral determinants associated with a mouse model of Langerhans cell histiocytosis (LCH), suppression subtractive hybridization (SSH) was used to identify unique viruses in the liver of severe combined immunodeficiency (SCID) mice transplanted with LCH tissues. A partial genomic sequence of a murine coronavirus was identified, and the whole genome (31428 bp) of the coronavirus was subsequently sequenced using PCR cloning techniques. Nucleotide sequence comparisons revealed that the genome sequence of the new virus was 91-93 % identical to those of known murine hepatitis viruses (MHVs). The predicted open reading frame from the nucleotide sequence encoded all known proteins of MHVs. Analysis at the protein level showed that the virus was closely related to the highly virulent MHV-JHM strain. The virus strain was named MHV-MI. No type D retroviruses were found. Degenerate PCR targeting of type D retrovirus and 5′-RACE targeting of other types of retroviruses confirmed the absence of any retroviral association with the LCH xenografted SCID mice.

## Introduction

Subtraction hybridization enables researchers to compare two populations of mRNA and to obtain clones of genes that are only expressed in one population. The basic principle behind the method is simple: both mRNA populations are converted to cDNA and then hybridized to each other; the hybridized sequences are then removed and the remaining unhybridized cDNAs represent genes that are only expressed in one population. Several different methods of subtraction hybridization have been successfully applied [1–4] but each had limitations in identifying rare transcripts. Suppression subtractive hybridization (SSH) [5] overcomes this by using an additional suppression PCR step that provides 10-100 fold enrichment of differentially expressed mRNAs, irrespective of their relative abundance. SSH has been successfully used to compare differences in gene expression between two transcriptomes [6–10] but, to our knowledge, the use of SSH for identification of an unknown virus has not been previously reported.

Langerhans cell histiocytosis (LCH) is a rare human disease of unknown etiology. The disease is characterized by the accumulation of clonally derived Langerhans cells [11, 12] and inflammatory cells including T cells, macrophages, eosinophils, neutrophils, giant cells, and plasma cells [13, 14]. The clinical representation of LCH can involve bone, skin, liver, spleen, lymph nodes, and/or bone marrow [15]. To overcome issues relating to the availability of LCH tissue, the establishment of a mouse model of LCH was attempted by xenografting human LCH tissue into severe combined immunodeficiency (SCID) mice [16]. Following engraftment, several mice developed pre-T cell lymphomas, from which a cell line was developed, called ThyE1M6. Electron microscopy of this cell line appeared to show a budding retrovirus with an appearance similar to that of type-D-like retroviral particles [16]. When ThyE1M6 cells were injected into SCID mice, a lethal syndrome developed within 2 weeks in which mice developed generalized granulomas involving inflammatory cells in multiple organs. A virus was suspected, but antiserum detection tests for known viruses were not possible, as SCID mice do not have B cells and therefore cannot make antibodies. Histopathological studies revealed that the liver was the most-affected organ. Attempts to isolate virus from liver were unsuccessful, and molecular techniques were used in this study to identify the putative virus.

A type-D retrovirus was initially suspected, based on EM morphology of the ThyE1M6 cell line and the biochemical readout of reverse transcriptase activity [16]. Subsequent to the original description of type D retroviral particles by Ristevski et al., other studies confirmed the existence of endogenous type D retroviral sequences in the mouse genome [17]. In this study, total RNA was extracted from infected and uninfected mouse liver, mRNA populations in total RNA were converted to cDNAs, and the cDNAs were subtracted from each other using the SSH method. The genomic sequence of a new strain of murine coronavirus was successfully identified and then sequenced. No type D retrovirus or any other type of retrovirus was found, even after using targeted degenerate PCR or 5′-RACE techniques. The new strain of coronavirus was named MHV-MI. To our knowledge, this is the first report of the isolation of an unknown viral sequence using the suppression subtractive hybridization method.

## Materials and methods

### Xenografting of SCID mice

A thymus biopsy sample from a 13-year-old female patient diagnosed with LCH was teased into a single-cell suspension and continuously cultured in the presence of 25 ng/ml (each) of TNF and GM-CSF (Boehringer Mannheim Biochemica, Mannheim, Germany) for 35 days. Live cells were purified on a Ficoll gradient and injected subcutaneously into each of three SCID mice along with TNF and GM-CSF. Cytokine injections were repeated daily for 5 days. Three additional SCID mice were injected with cytokines only as the control. Liver tissues were harvested from the mice after 7 weeks for histopathologic examination and then stored at -80 °C for future molecular biological studies. This work was approved by RCH (Royal Children Hospital) Ethics in Human Research and RCH Animal Experimentation Ethics Committee under the project title ‘The Biology of Langerhans Cell Histiocytosis’.

### RNA extraction

Total RNA was extracted from approximately 50 µg of frozen liver tissues or 1 × 10^6^ ThyE1M6 cells using a miRNeasy Kit (QIAGEN, Clifton Hill, Australia) and following the manufacturer’s instruction manual. A minor modification was made in the final stage of the protocol where RNA was eluted with warm DEPC-treated water (50 °C) and collected in an Eppendorf tube containing 1 µl of RNasin (Promega, Madison, USA) and1 µl of DTT (10 mM).

### cDNA synthesis

A BD SMART RACE cDNA Amplification kit (BD Biosciences-Clontech, Palo Alto, USA) was used for the synthesis of first-strand cDNA from total RNA extracted from mouse liver tissues or ThyE1M6 cells. The protocol in the user manual was slightly modified to adjust the reagent volume. Two micrograms of total RNA (2-4 µl), 0.5 µl of 5′-RACE CDS primer (12 µM), 0.5 µl of SMART II Oligonucleotide (12 µM) and deionized water to 5 µl final volume were added together in a sterile PCR reaction tube (0.1 ml). Contents were mixed and briefly spun. The tube was incubated at 72 °C in a hot-lid thermal cycler for 2 min and then cooled down on ice for 2 min. A mixture of 5× First-Strand Buffer (2 µl), 10 mM dNTP mix (1 µl), 20 mM DTT (1 µl), and Powerscript Reverse Transcriptase (1 µl) was added to the tube and mixed gently by tapping. The mixture was briefly spun down and incubated at 42 °C for 4 h (enough time to transcribe full-length type D retroviral RNA). After the reaction was finished, the transcriptase activity was stopped by incubating the tube at 70 °C for 7 min. The transcribed cDNA was then either stored at -20 °C for future use or used immediately.

### Preparation of forward subtracted cDNA by SSH

The preparation of forward subtracted cDNAs was done according to the BD PCR-Select cDNA Subtraction Kit (BD Biosciences-Clontech, Palo Alto, USA) protocol. cDNAs were synthesized from total RNA of both infected and uninfected mouse liver using the protocol described above. cDNA from infected mouse liver was called ‘tester cDNA’, and that from uninfected mouse liver was called ‘driver cDNA’.

Both cDNAs were then purified using Chroma Spin -400 column chromatography (QIAGEN, Clifton Hill, Australia) and digested with *Rsa* I restriction enzyme (kit component) to completion. Confirmation of complete digestion was monitored by gel electrophoresis. Digested cDNAs were then purified using the NucleoSpin II protocol (BD Biosciences-Clontech, Palo Alto, USA).

Digested and purified tester cDNA was subdivided into two equal portions and ligated with two different adaptors (supplied in the cDNA subtraction kit). Ligation efficiency was confirmed by PCR using a housekeeping gene (G3PDH)-specific forward primer and the reverse adaptor primer (kit components). Following ligation, two hybridization steps were performed as per the protocol. Normalized and subtracted single-stranded target cDNA molecules anneal with each other, forming double-stranded hybrids with two different adaptor sequences at their 5′ ends. The adaptor ends were then filled with DNA polymerase, and the subtracted molecules were specifically amplified by nested PCR using adaptor-specific primer pairs.

### Preparation of subtracted cDNA libraries

Subtracted cDNAs (the nested PCR products described in the previous section) were treated with *Taq* DNA polymerase for 10 min at 72 °C in a reaction mix of 20 µl containing 17 µl of nested PCR products, 2 µl of PCR buffer (×10), 0.5 µl of MgCl_2_, 0.25 µl of dNTP mix, and 0.25 µl of *Taq* DNA polymerase (Invitrogen, California, USA). Two µl of treated cDNAs was then ligated with 25 ng of pGEM-T plasmid vector (Promega, Madison, USA) using T4 DNA ligase in an overnight reaction at 16 °C. Ligase activity was then stopped by heating the reaction mix at 72 °C for 10 min. One µl of inactive ligation mix was then used to transform 50 µl of maximum efficiency *E. coli* (DH5α) competent cells (Invitrogen, Carlsbad, USA) using a MicroPulser (BIO-RAD, Hercules, USA). A sufficient volume of transformed bacteria was plated onto LB agar plates containing 50 µg of ampicillin (Aspen, Deakin, Australia) per ml and an appropriate amount of Xgal (Promega, Madison, USA) and IPTG (Invitrogen, Carlsbad, USA), followed by overnight incubation at 37 °C. Multiple plates were grown from each ligation mix. The pGEM-T plasmid contains a LacZ reporter at the multiple cloning site and allows blue/white screening. White colonies represented recombinant cells containing a subtracted cDNA insert, while blue colonies represented background cells containing plasmids only. Fully grown colonies (blue and white) from each plate were collected with a scrubber in 5 ml of LB + 25 % glycerol and pooled in a 50-ml tube. Preparation of the subtracted cDNA library was then complete. The library was divided into several vials, flash frozen with liquid nitrogen, and stored at -80 °C for future use.

### Titering and sequencing forward subtracted cDNA clones

One vial of frozen forward subtracted library was quickly defrosted on a warm (37 °C) metal block rack, mixed by gentle vortexing, and then diluted 10^−3^-fold and 10^−6^-fold with LB broth. Ten µl of each diluted library was mixed with 100 µl of LB broth and inoculated onto LB plates containing 50 µg of ampicillin per ml and the appropriate amount of Xgal (Promega, Madison, USA) and IPTG (Invitrogen, Carisbad, USA). Plates were incubated overnight at 37 °C. A satisfactorily grown plate was selected, and both white and blue cells were counted. The titer of the library was calculated from the white colony counts, and the efficiency of the library was calculated from the ratio of white cell counts and total cell counts.

A total of 148 white colonies were randomly selected from a titering plate and grown in 2 ml of LB-ampicillin medium overnight at 37 °C with vigorous shaking. Recombinant plasmids were prepared from all colonies using the QIAprep^®^ procedure (QIAGEN, Clifton Hill, Australia). Sequencing reactions were carried out using PRISM BigDye Terminator Mix (Applied Biosystems, Foster City, USA) and vector-specific primers. A 3730s Genetic Analyzer (Applied Biosystems, Foster City, USA) was used for reading sequences. The sequences were then identified based on homology searches using the web-based nucleotide sequence analysis program MegaBLAST [18] from the National Center for Biotechnology Information (NCBI).

### Primer design and sequencing of the MHV-MI virus genome

GeneFisher2 software (Bielefeld University Bioinformatics Server – BiBiServ) was used to design 34 pairs of overlapping primers, each covering approximately 1000 bp of the MHV-A59 genomic sequence (accession no. AY700211). Primers were designed to enable a single thermal profile to be used for each PCR. Twenty-three pairs (Table 1, yellow shaded) produced the expected PCR products with a thermal profile of 5 min pre-PCR treatment at 96 °C; 35 cycles of 95 °C for 15 s, 55 °C for 30 s and 72 °C for 1 min, followed by a 5-min extra elongation at 72 °C. The PCR cocktail (20 µl) contained 1 µl of fivefold-diluted cDNA from the infected mouse (E4M31) liver, 2.5 mM MgCl_2_, 0.5 µl of each primer (10 µM), 0.5 µl of dNTP mix (10 mM), 0.2 µl of Advantage^®^ 2 Polymerase Mix (Clontech, CA 94043, USA), and the required volume of water. PCR products were cloned and sequenced using a previously established protocol [19]. A 3730s Genetic Analyzer was used instead of a 310 Genetic Analyzer in the new method.

**Table 1:**
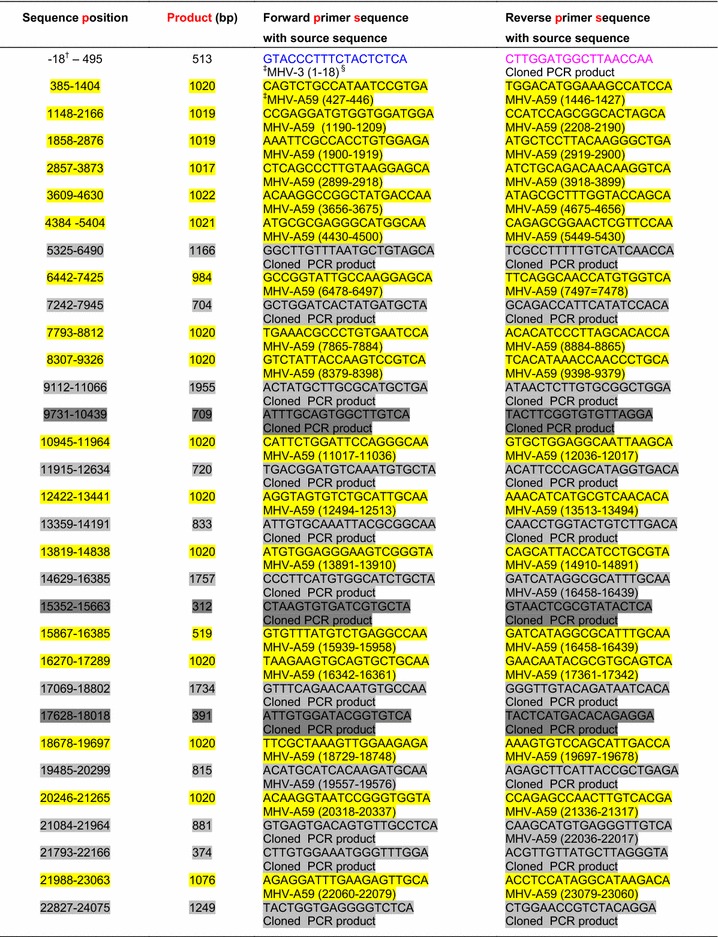

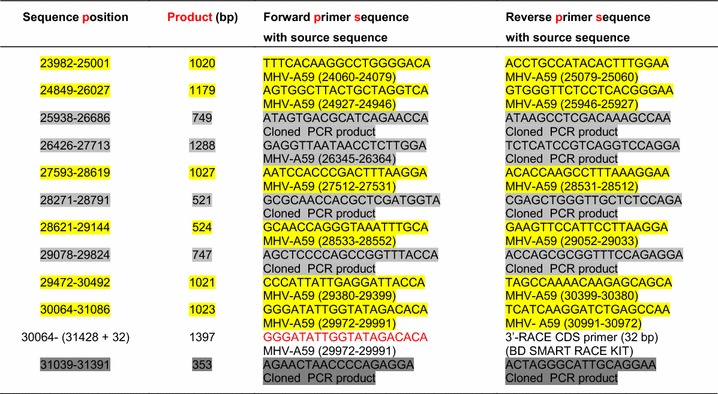
Primer list for cloning and sequencing the full-length MHV-MI virus genome

^†^ Negative sequence position means the position before the start of the reported sequence

^‡^ The accession number for the MHV-3 genome sequence is FJ647224.1, and that for the MHV-A59 genome sequence is AY700211.1

^§^ The number range within the brackets denotes the nucleotide positions in the respective genome sequence

In the second phase, 11 pairs of nested primers located within the sequences of previously cloned PCR products and four pairs of combined primer (nested and MHV-A59 genome-specific) were designed (Table 1, gray shaded). Varying thermal profiles were used depending on the melting temperatures of primer pairs. All 16 PCR products were cloned and sequenced again.

In the third phase, the 3′ end of the genome was amplified by 3′-RACE reaction using a BD SMART RACE cDNA Amplification Kit (BD Biosciences-Clontech, Palo Alto, USA) and an internal primer (5′-GGGATATTGGTATAGACACA-3′) that was designed from the MHV-A59 genomic sequence (Table 1, red letters). The 5′ end of the genome was amplified by standard PCR using a known 5′-end conserved primer (5′-GTACCCTTTCTACTCTCA-3′) (Table 1, blue letters) and an internal reverse primer (5′-CTTGGATGGCTTAACCAA-3′) (Table 1, pink letters), which was designed from the 5′-end of the already sequenced genome portion.

In the fourth phase, four pairs of nested primers (Table 1, dark gray shaded) were designed to further sequence the less-reliable middle portion of longer sequences (longer than 1300 bp). PCR products were amplified using varying thermal profiles depending on the melting temperatures of primer pairs. Finally, a total of 44 primer pairs were used to amplify the full-length viral genome (Table 1).

All PCR products or RACE products were cloned and sequenced using the same method [19]. Compilation and further analysis of those sequences were performed using pDRAW32 DNA Analysis Software (http://www.acaclone.com).

### Degenerate PCR

#### Designing degenerate primers and optimization of PCR conditions

Nucleotide sequences of almost all known Type-D retroviruses, namely SRV-1, SRV-2, MPMV, Tsukuba monkey virus, squirrel retrovirus, TvERV, MusD1 and MusD2 [17, 20–26], were aligned using the web-based CLUSTAL W (1.83) program (EMBL-EBI). Two pairs of degenerate primers each from the ‘gag’, ‘pol’ and ‘pro’ regions of the viral sequence were designed from the conserved regions of the alignment data (not shown here). The primer pairs used were as follows:

gag-PP1-FWD: 5′-TTRGCRTTYTCAWADGCYA-3′
gag-PP1-REV: 5′-AITAYGGBSCYWMWGCYCC-3′
gag-PP2-FWD: 5′-TRGCHTWTGARAAYGCYAA-3′
gag-PP2-REV: 5′-GRCCCCKBHIBHMGTTTCC-3′

pol-PP1-FWD: 5′-HATWGAYYTIAARGAYTGY-3′
pol-PP1-REV: 5′-RIARYTTTTGRAARTCATT-3′
pol-PP2-FWD: 5′-IYTRGGWGAYATHAATTGG-3′
pol-PP2-REV: 5′-GARGABCCRTCWGTRAADA-3′

pro-PP1-FWD: 5′-GABTGGACYTSWGTKCCAC-3′
pro-PP1-REV: 5′-TRAWYTCYCCHKYATARTC-3′
pro-PP2-FWD: 5′-GRGARWTYAARATHWTDGC-3′
pro-PP2-REV: 5′-CYCCIGWRTCKAKTAVBCC-3′

(where R = A+G, Y = C+T, M = A+C, K = G+T, S = G+C, W = A+T, H = A+T+C, B = G+T+C, D = G+A+T, V = G+A+C, I = Inosine)

Optimization of PCR conditions for all degenerate primer pairs was done in two phases using a mouse genomic DNA template containing the type D retroviral sequences MusD1 and MusD2 [17]. In the first phase, only one thermal profile (95 °C for 7 min, 40 cycles of 95 °C for 30 s, 40 °C for 10 s, 60 °C for 1 min, and an additional 5-min extension at 60 °C) was used for all six primer pairs. A standard PCR mix containing 2 mM MgCl_2_ was used to amplify the target PCR products in this phase. In the second phase, only the successful primer pairs in the first phase (gag-PP2-FWD/REV and pro-PP2-FWD/REV) were optimized, using various MgCl_2_ concentrations and annealing temperatures.

#### Experimental degenerate PCR

Experimental degenerate PCR reactions were carried out with the cDNAs obtained from infected and uninfected mouse liver. Only successfully optimized primer pairs were used to amplify the target PCR products under the optimized thermal conditions.

### Internal control RT-PCR

A predesigned primer pair for the mouse beta actin gene with PrimerBank ID 6671509a1 (http://pga.mgh.harvard.edu/primerbank/) was used to amplify an RT-PCR product from the cDNA of both infected and uninfected mouse liver. The reaction mix (20 µl) contained 1 µl of cDNA, 2.5 mM MgCl_2_, 0.5 µl of each primer (10 µM), 0.5 µl of dNTP mix (10 mM), 2 units of AmpliTaq Gold DNA polymerase (Applied Biosystems, Foster City, USA) and the required volume of water to make the final volume 20 µl. The thermal profile used was 95 °C for 7 min, 40 cycles of 95 °C for 30 s, 55 °C for 10 s, and 60 °C for 30 s, followed by an additional 5-min extension at 60 °C.

### 5′- RACE using tRNA primers

Eleven tRNA primers complementary to the primer binding sites (PBS) of most of the known retroviral genomes (Table 2), were synthesized by Sigma-Aldrich (Australia). 5′-RACE reactions were carried out with all of these primers against the cDNA obtained from the infected mouse (E4M31) liver according to the BD SMARTTM RACE cDNA Amplification Kit protocol (BD Bioscience-Clontech, Palo Alto, CA, USA). A positive-control 5′-RACE was done with the cDNA obtained from the ThyE1M6 cell line using t-RNA Pro primer. Reactions were done in a 25-µl final volume containing first-strand cDNA (2 µl), 10× PCR buffer (2.5 µl), 25 mM MgCl_2_ (2.0 µl), 10 mM dNTP mix (0.5 µl), 10 µM t-RNA primer (0.5 µl), universal primer mix (UPM) (2 µl), AmpliTaq Gold DNA polymerase (Applied Biosystems, Foster City, USA) (0.5 µl) and water (remaining volume up to 25 µl). The thermal profile used for the 5′-RACE reaction was 5 min pre-PCR treatment at 96 °C; 35 cycles of 95 °C for 15 s, 48 °C for 30 s and 72 °C for 30 s; and 5 min extra elongation at 72 °C.

**Table 2:** Retroviral PBS sequences

Virus names	Common PBS sequence	PBS type
TvERV, HERV, M-PMV, SFV	5′-tggcgcccaagctggggc-3′	C-tRNA^†^ Lys
HIV-1, MMTV, SIV, SRV-1	5′-tggcgcccgaacagggac-3′	C-tRNA Lys-3
S-HIV mutant	5′-tggcgccctgaacagggac-3′	C-tRNA Lys-5
FMLV, RMLV, MCRV, STLV-3, STLV-1, HTLV-1, Abelson MLV, Mo-MLV, MOSV, KoRV	5′-tgggggctcgtccgggat-3′	C-tRNA Pro
Mu-ERVL	5′-tggaggtcccacgagat-3′	C-tRNA Thr
MLV	5′-tggaggcccagcgagat-3′	C-tRNA Gln
ALV, SRV, ACV, EAV-HP	5′-tggtgaccccgacgtgat-3′	C-tRNA Trp
AKV	5′-tggcgctgcgagcaggac-3′	C-tRNA Ser
AKV	5′-tgccaccccagacgggac-3′	C-tRNA Arg
AKV	5′-tggtgcctaaacccggga-3′	C-tRNA Phe
MusD1, MusD2^††^	5′-tggcgccagaactgggac-3′	C-tRNA Lys-5v

^†^ C-tRNA, complementary to tRNA

^††^ MusD1 and MusD2 are not true viruses but are endogenous proviruses found in the mouse genome [17]

## Results

### Identification of MHV sequences using the SSH technique

Subtraction efficiency tests for control and experimental samples were performed after completion of the forward subtraction process, and the results were satisfactory (data not shown). Successfully subtracted cDNAs were then cloned into *E. coli* using the pGEM vector and made into a forward subtracted cDNA library following the procedure described above. The titer and efficiency of the library were calculated to be 4.3 × 10^9^ cfu/ml and 71.5 %, respectively. Sequence analysis of 148 successful clones revealed that 12 % of the total subtracted genes belonged to murine coronavirus (MHV) (Table 3). No other viral genes were identified in the subtracted population (148 clones). This indicated that murine hepatitis virus was a likely cause of the infection in transplant-recipient SCID mouse liver.

**Table 3:** Gene list from forward cDNA subtraction

HITS	Percentage	Gene name
18	12	Murine hepatitis virus nucleocapsid, membrane proteins etc.
14	9	Mouse serum amyloid A (SAA) family proteins
14	9	Mouse fibrinogen (alpha, beta and gamma) polypeptide
6	4	Mouse annexin A protein family
3	2	Mouse lactotransferrin
2	1.33	Mouse serine or cysteine peptidase inhibitor (SP1-2) clade member
2	1.33	Mouse mRNA for Ly-6 alloantigen (ly-6E.1)
1	0.68	89 individual mouse genes

### Sequencing the full-length murine coronavirus (MHV) genome

A total of 43 PCR products of varying sizes (312 bp-1757 bp) and a 3′-RACE product (1365 bp) were sequenced on both strands to derive the full-length genome sequence of the murine hepatitis virus. At least three clones from each PCR product or 3′-RACE product were sequenced. DNA analysis software pDRAW32 and ClustalW2 (EMBL-EBI) were use to compile and align sequence data. Sequences in the primer regions were corrected using overlapping sequence alignment. Since the sequence of the 5′-end forward primer (5′GTACCCTTTCTACTCTCA3′) could not be verified by any other means, an 18-bp sequence from the 5′ end of the full-length genome was excluded and then reported to DNA Data Bank of Japan (DDBJ) with the accession number AB551247. The reported full-length genome of MHV-MI is therefore 31,428 bp long.

### Sequence analysis

Initially 12 ORFs were predicted in three reading frames from the full-length viral genome (31,428 bp) using pDRAW32 DNA analysis software (Aca Clone software). Only five of these matched with known proteins of MHVs: hemagglutinin-esterase (HE), spike glycoprotein (S), non-structural protein 4 (ns4), membrane protein (M), and nucleocapsid protein (NC). The remaining seven ORFs were either partially matched or did not match with any known MHV proteins. At that stage, the information related to ORFs of many MHVs with published [27–33] or unpublished sequences (accession nos. FJ647224, FJ647219, NC_006852.1) were consulted, and seven more proteins, namely, replicase polyprotein 1a, replicase polyprotein 1ab, non-structural protein 2a (ns2a), non-structural protein 5 (ns5), envelope protein (E), and internal protein (I) were predicted. The ORF of replicase polyprotein 1ab contained a ribosomal slippage site at nucleotide position 13,524 bp. ORFs of replicase polyprotein 1ab, replicase polyprotein 1a, ns4, ns5, E, M, NC and I protein contained overlapping sequences of varying length (4–13384 bp). The I protein was predicted within the coding sequence of the NC protein, but in a different reading frame; hence, there was 100 % overlap (410 bp) of the I protein by the NC protein. BLAST analysis against the protein data base revealed that most of the predicted proteins are more similar to the corresponding proteins of MHV-3 (90-99 %) than to those of any other MHV strain (Table 4). Comparative analysis of ORFs from different MHVs revealed that MHV-MI contains the same number of proteins as JHM virus or its variants RJHM/A, MHV-JHM.IA, SA59/RA59, SJHM/RA59, and strain A59 (Table 4), and hence, MHV-MI is more closely related to JHM virus or its variants. The reported full-length genome sequence of MHV-MI in the DDBJ database (accession no. AB551247) contained all of these 12 predicted ORFs and their nucleotide positions.

**Table 4:** Sequence identity of viral proteins to those of other MHV viruses

Virus name	Size	ORF1ab	ORF1a	ns2a	HE	S	ns4	ns5	EP	M	N	I
MHV-3	31,448	99	97	97	0^†^	96	0	90	96	99	97	90
MHV-1	31,386	99	97	96	0	90	94	89	95	99	96	0
MHV-2 / MHV-ML11	31,233	98	94	95	0	90	94	87	96	99	95	0
Strain JHM	31,526	98	95	95	81	95	94	90	95	97	96	89
RJHM/A	31,427	98	96	94	82	95	94	90	95	98	96	90
MHV-JHM.IA	31,473	99	96	94	81	95	94	92	96	98	96	90
Strain A59	31,335	99	96	96	81	90	96	92	93	99	96	0
SA59/RJHM	31,283	98	96	94	81	91	94	90	95	98	96	89
SJHM/RA59	31,250	99	96	94	81	90	0	92	93	99	96	89

^†^ 0, protein absent in the target virus

### Degenerate PCR targeting type D retrovirus

**Fig. 1:**
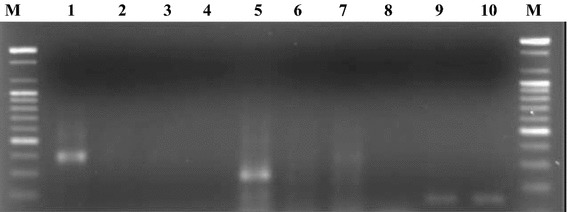
Degenerate PCR with optimized primer pairs, gag-PP2-FWD/REV and pro-PP2-FWD/REV. Under the optimized conditions, the gag-PP2-FWD/REV primer pair produced a 420-bp PCR product from mouse genomic DNA (lane 1) but not from the cDNA from infected mouse liver (lane 2) or uninfected mouse liver (lane 3). Similarly, the pro-PP2-FWD/REV primer pair produced a 240-bp PCR product from mouse genomic DNA (lane 5) but not from the cDNA of infected mouse liver (lane 6) or uninfected infected mouse liver (lane 7). Lanes 4 and 8 represent negative controls (no template) for each primer. Lanes 9 and 10 show the RT-PCR products (154 bp) from infected and uninfected mouse liver cDNAs, respectively, using β-actin gene primers. Lane M shows a 100-bp ladder marker, and the lowest band represents 200 bp

Primers for degenerate PCR targeting type D retrovirus were designed from the known sequences of type D retroviruses as described above. After the first PCR optimization step, only two primer pairs (gag-PP2-FWD/REV and pro-PP2-FWD/REV) successfully amplified the target PCR products of 420 bp and 240 bp, respectively, from the mouse genomic DNA. PCR products were verified by sequencing to be derived from MusD1 or MusD2 retroviral sequences of mouse genomic DNA. After the second phase of optimization, 2 mM MgCl_2_ was found to be optimal for both primer pairs, but an annealing temperature of 55 °C was found to be optimum for the gag- PP2-FWD/REV primer pair, and 45 °C for the pro-PP2-FWD/REV primer pair. Under the optimum conditions, both primer pairs produced sharp PCR bands (Fig. 1). In the experimental degenerate PCR, no PCR products were amplified, even after 70 cycles. RT-PCR internal control with mouse β-actin primers produced the expected products (154 bp) from both infected and uninfected mouse liver cDNAs. These results clearly indicated that no type D retrovirus was present in the infected or uninfected mouse liver.

### 5′- RACE targeting a whole range of retroviruses

**Fig. 2:**
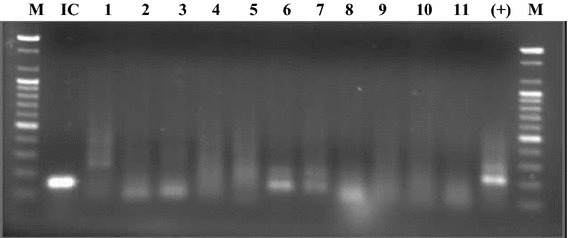
5′-RACE using tRNA primers. Eleven tRNA primers were used to produce the expected 5′-RACE product from the infected mouse (E4M31) liver cDNA as per the procedure described in Materials and methods. Lane 1, Lys tRNA; lane 2, Lys-3 tRNA; lane 3, Lys-5 tRNA; lane 4, Lys-5v tRNA; lane 5, Pro tRNA; lane 6, Thr tRNA; lane 7, Gln tRNA; lane 8, Trp tRNA; lane 9, Ser tRNA; lane 10, Arg tRNA, and lane 11, Phe tRNA. Lane- (+) shows a positive control 5′-RACE product (220 bp) with the cDNA of the ThyE1M6 cell line using Pro tRNA. Lane IC shows an internal control RT-PCR product (154 bp) from the cDNA of infected mice liver, using β-actin primers. Lane M shows a 100-bp ladder marker

Eleven tRNA primers were designed to cover the whole range of retroviruses (Table 2), but none of the primers produced the expected 5′-RACE products from cDNA from infected mouse liver (Fig. 2). tRNA primers for Lys, Thr and Gln produced some products (lanes 1, 6 and 7), but similar products were also observed in the 5′-RACE experiment with uninfected mouse liver cDNA (data not shown). These products were regarded as not being generated from any retroviral genome. A positive control cDNA from the ThyE1M6 cell line, which expresses endogenous MoMLV (data not shown), yielding a product with the tRNA-Pro primer (Fig. 2). The product was also confirmed by sequencing. An RT-PCR internal control with mouse β-actin gene primers, following the procedure described above, produced the expected product from infected mouse liver cDNA (Fig. 2). These results indicated that no known retrovirus was present in the infected mouse liver.

## Discussion

The PCR-Select^™^ cDNA subtraction method is based on a unique approach of selective amplification of differentially expressed sequences [5, 34] and requires a series of quality-assessment steps before the final subtraction stage. We started with three pairs of infected and uninfected mouse liver RNA samples, and one pair of positive control human placental RNA sample (HaeIII treated and untreated). After quality testing in each step of cDNA synthesis, *Rsa* I digestion, and adaptor ligation, only one pair of ‘tester cDNA’ and ‘driver cDNA’ was selected for final subtraction analysis, based on quality assessment. The ‘tester cDNA’ was derived from the RNA of infected mouse (E4M31) liver, and the driver cDNA was derived from RNA of uninfected mouse (E5M32) liver. Since identification of a viral gene signature from the infected mouse liver was the goal, only the forward subtraction method was used, and the library was constructed from the forward-subtracted cDNAs only. Other gene sequences resulting from forward subtraction analysis (Table 2) were not analyzed for the same reason.

MHVs belong to the group II coronaviruses, which are divided into two groups according to patterns of tropism [35]. Enterotropic strains, such as MHV-D, MHV-Y, MHV-RI, LIVIM, and DVIM, generally produce infections confined to the GI tract [36], whereas polytropic strains, such as MHV-1, MHV-2, MHV-3, MHV-4 (or JHM), MHV-A59 and MHV-S, initiate infection mainly in the respiratory tract, which can disseminate to the liver, spleen, lymph nodes, and brain [35–37]. Polytropic strains are usually more virulent than enterotropic strains [38]. Although BLAST analysis of protein sequences showed a higher percentage of homology with MHV-3 proteins, comparative analysis of ORFs (Table 4) revealed that MHV-MI was more closely related to polytropic MHV-JHM virus or its variants. MHV-MI also contained the HE protein, which forms smaller spikes on virions [27] and enhances virulence when paired with spike protein (S) [39]. The HE protein is absent in the MHV-3 strain, and MHV-MI could therefore be predicted to be a polytropic virulent virus and a very close relative of MHV-JHM.

The non-specific SSH approach is useful for identifying unknown putative viruses. Since it did not identify the suspected type D retrovirus or any other retroviruses, targeted degenerate PCR and 5′-RACE techniques were used to confirm the negative SSH results. Both experiments confirmed the absence of any retroviral involvement in the diseased mouse livers. It is unclear if the inflammatory LCH-like phenotype observed in the SCID mice is related to the MHV infection, and further studies are required to examine this possibility.

## Abbreviations

EM: Electron microscopy
TNF: Tumor necrosis factor
GM-CSF: Granulocyte macrophage-colony stimulating factor
DTT: Dithiothreitol
G3PDH: Glyceraldehyde-3-phosphate dehydrogenase
IPTG: Isopropylthio-β-d-galactoside
DEPC: Diethylpyrocarbonate
LB: Luria-Bertani
ORF: Open reading frame

## References

[1] Sargent TD, Dawid IB (1983). Differential gene expression in the gastrula of Xenopus laevis. Science.

[2] Hedrick SM, Cohen DI, Nielsen EA, Davis MM (1984). Isolation of cDNA clones encoding T cell-specific membrane-associated proteins. Nature.

[3] Duguid JR, Dinauer MC (1990). Library subtraction of in vitro cDNA libraries to identify differentially expressed genes in scrapie infection. Nucl Acids Res.

[4] Hara E, Kato T, Nakada S, Sekiya S, Oda K (1991). Subtractive cDNA cloning using oligo(dT)30-latex and PCR: isolation of cDNA clones specific to undifferentiated human embryonal carcinoma cells. Nucl Acids Res.

[5] Diatchenko L, Lau YF, Campbell AP, Chenchik A, Moqadam F, Huang B, Lukyanov S, Lukyanov K, Gurskaya N, Sverdlov ED, Siebert PD (1996). Suppression subtractive hybridization: a method for generating differentially regulated or tissue-specific cDNA probes and libraries. Proc Natl Acad Sci USA.

[6] Kuang WW, Thompson DA, Hoch RV, Weigel RJ (1998). Differential screening and suppression subtractive hybridization identified genes differentially expressed in an estrogen receptor-positive breast carcinoma cell line. Nucl Acids Res.

[7] Patzwahl R, Meier V, Ramadori G, Mihm S (2001). Enhanced expression of interferon-regulated genes in the liver of patients with chronic hepatitis C virus infection: detection by suppression-subtractive hybridization. J Virol.

[8] Kiss C, Nishikawa J, Dieckmann A, Takada K, Klein G, Szekely L (2003). Improved subtractive suppression hybridization combined with high density cDNA array screening identifies differentially expressed viral and cellular genes. J Virol Methods.

[9] Shackel NA, McGuinness PH, Abbott CA, Gorrell MD, McCaughan GW (2003). Novel differential gene expression in human cirrhosis detected by suppression subtractive hybridization. Hepatology.

[10] Munir S, Singh S, Kaur K, Kapur V (2004). Suppression subtractive hybridization coupled with microarray analysis to examine differential expression of genes in virus infected cells. Biol Proc Online.

[11] Willman CL, Busque L, Griffith BB, Favara BE, McClain KL, Duncan MH, Gilliland DG (1994). Langerhans’-cell histiocytosis (histiocytosis X)–a clonal proliferative disease. N Engl J Med.

[12] Yu RC, Chu C, Buluwela L, Chu AC (1994). Clonal proliferation of Langerhans cells in Langerhans cell histiocytosis. Lancet.

[13] Chu T, D’Angio GJ, Favara B, Ladisch S, Nesbir M, Prichard J (1987). Histiocytosis syndromes in children. Lancet.

[14] Kannourakis G, Abbas A (1994). The role of cytokines in the pathogenesis of Langerhans cell histiocytosis. Br J Cancer Suppl.

[15] Jaffe R, DeVaughn D, Langhoff E (1998). Fascin and the differential diagnosis of childhood histiocytic lesions. Pediatr Dev Pathol.

[16] Ristevski S, Purcell DF, Marshall J, Campagna D, Nouri S, Fenton SP, McPhee DA, Kannourakis G (1999). Novel endogenous type D retroviral particles expressed at high levels in a SCID mouse thymic lymphoma. J Virol.

[17] Mager DL, Freeman JD (2000). Novel mouse type D endogenous proviruses and ETn elements share long terminal repeat and internal sequences. J Virol.

[18] Morgulis A, Coulouris G, Raytselis Y, Madden TL, Agarwala R, Schaffer AA (2008). Database indexing for production MegaBLAST searches. Bioinformatics.

[19] Islam MM (2008) Molecular cloning, expression and characterization of a serine proteinase from Japanese edible mushroom, *Grifola frondosa*: solving the structure–function anomaly of a reported aminopeptidase. Elect J Biotechnol. ISSN: 0717-3458. doi:10.2225/vol11-issue4-fulltext-5-. http://www.ejbiotechnology.info/vol11/issue4/full/5/index.html

[20] Chiu IM, Callahan R, Tronick SR, Schlom J, Aaronson SA (1984). Major pol gene progenitors in the evolution of oncoviruses. Science.

[21] Power MD, Marx PA, Bryant ML, Gardner MB, Barr PJ, Luciw PA (1986). Nucleotide sequence of SRV-1, a type D simian acquired immune deficiency syndrome retrovirus. Science.

[22] Sonigo P, Barker C, Hunter E, Wain-Hobson S (1986). Nucleotide sequence of Mason-Pfizer monkey virus: an immunosuppressive D-type retrovirus. Cell.

[23] Thayer RM, Power MD, Bryant ML, Gardner MB, Barr PJ, Luciw PA (1987). Sequence relationships of type D retroviruses which cause simian acquired immunodeficiency syndrome. Virology.

[24] York DF, Vigne R, Verwoerd DW, Querat G (1992). Nucleotide sequence of the jaagsiekte retrovirus, an exogenous and endogenous type D and B retrovirus of sheep and goats. J Virol.

[25] Baillie GJ, Wilkins RJ (2001). Endogenous type D retrovirus in a marsupial, the common brushtail possum (Trichosurus vulpecula). J Virol.

[26] Hara M, Sata T, Kikuchi T, Nakajima N, Uda A, Fujimoto K, Baba T, Mukai R (2005). Isolation and characterization of a new simian retrovirus type D subtype from monkeys at the Tsukuba Primate Center, Japan. Microbes Infect.

[27] Luytjes W, Bredenbeek PJ, Noten AF, Horzinek MC, Spaan WJ (1988). Sequence of mouse hepatitis virus A59 mRNA 2: indications for RNA recombination between coronaviruses and influenza C virus. Virology.

[28] Gombold JL, Hingley ST, Weiss SR (1993). Fusion-defective mutants of mouse hepatitis virus A59 contain a mutation in the spike protein cleavage signal. J Virol.

[29] Das Sarma J, Fu L, Hingley ST, Lai MM, Lavi E (2001). Sequence analysis of the S gene of recombinant MHV-2/A59 coronaviruses reveals three candidate mutations associated with demyelination and hepatitis. J Neurovirol.

[30] Navas S, Weiss SR (2003). Murine coronavirus-induced hepatitis: JHM genetic background eliminates A59 spike-determined hepatotropism. J Virol.

[31] Ontiveros E, Kim TS, Gallagher TM, Perlman S (2003). Enhanced virulence mediated by the murine coronavirus, mouse hepatitis virus strain JHM, is associated with a glycine at residue 310 of the spike glycoprotein. J Virol.

[32] Coley SE, Lavi E, Sawicki SG, Fu L, Schelle B, Karl N, Siddell SG, Thiel V (2005). Recombinant mouse hepatitis virus strain A59 from cloned, full-length cDNA replicates to high titers in vitro and is fully pathogenic in vivo. J Virol.

[33] De Albuquerque N, Baig E, Ma X, Zhang J, He W, Rowe A, Habal M, Liu M, Shalev I, Downey GP, Gorczynski R, Butany J, Leibowitz J, Weiss SR, McGilvray ID, Phillips MJ, Fish EN, Levy GA (2006). Murine hepatitis virus strain 1 produces a clinically relevant model of severe acute respiratory syndrome in A/J mice. J Virol.

[34] Gurskaya NG, Diatchenko L, Chenchik A, Siebert PD, Khaspekov GL, Lukyanov KA, Vagner LL, Ermolaeva OD, Lukyanov SA, Sverdlov ED (1996). Equalizing cDNA subtraction based on selective suppression of polymerase chain reaction: cloning of Jurkat cell transcripts induced by phytohemaglutinin and phorbol 12-myristate 13-acetate. Anal Biochem.

[35] Barthold SW, Smith AL (1984). Mouse hepatitis virus strain—related patterns of tissue tropism in suckling mice. Arch Virol.

[36] Homberger FR, Zhang L, Barthold SW (1998). Prevalence of enterotropic and polytropic mouse hepatitis virus in enzootically infected mouse colonies. Lab Anim Sci.

[37] Weiss SR, Navas-Martin S (2005). Coronavirus pathogenesis and the emerging pathogen severe acute respiratory syndrome coronavirus. Microbiol Mol Biol Rev.

[38] Fujiwara K, Wagner JE, Waggie K, Kagiyama N, Allen M (1994). Mouse hepatitis virus. Manual of microbiologic monitoring of laboratory animals.

[39] Kazi L, Lissenberg A, Watson R, de Groot RJ, Weiss SR (2005). Expression of hemagglutinin esterase protein from recombinant mouse hepatitis virus enhances neurovirulence. J Virol.

